# A theoretical perspective on action consequences in action imagery: internal prediction as an essential mechanism to detect errors

**DOI:** 10.1007/s00426-023-01812-0

**Published:** 2023-03-24

**Authors:** Martina Rieger, Shaun G. Boe, Tony G. J. Ingram, Victoria K. E. Bart, Stephan F. Dahm

**Affiliations:** 1 Institute for Psychology, UMIT Tirol—Private University for Health Sciences and Health Technology, Eduard Wallnöfer Zentrum 1, 6060 Hall in Tyrol, Austria; 2 Laboratory for Brain Recovery and Function, School of Physiotherapy, Dalhousie University, Nova Scotia, Canada; 3 Faculty of Psychology and Sports Science, Department of Psychology, Universität Innsbruck, Innsbruck, Austria

## Abstract

Acting in the environment results in both intended and unintended consequences. Action consequences provide feedback about the adequacy of actions while they are in progress and when they are completed and therefore contribute to monitoring actions, facilitate error detection, and are crucial for motor learning. In action imagery, no actual action takes place, and consequently, no actual action consequences are produced. However, imagined action consequences may replace actual action consequences, serving a similar function and facilitating performance improvements akin to that occurring with actual actions. In this paper, we conceptualize action imagery as a simulation based on internal models. During that simulation, forward models predict action consequences. A comparison of predicted and intended action consequences sometimes indicates the occurrence of action errors (or deviations from optimal performance) in action imagery. We review research indicating that action errors are indeed sometimes imagined in action imagery. These results are compatible with the view that action imagery is based on motor simulation but incompatible with the view that action imagery is solely based on abstract knowledge. The outlined framework seems suitable to cover a wide range of action imagery phenomena and can explain action imagery practice effects.

## Introduction

Imagine yourself in a gymnasium, standing at the free-throw line holding a basketball in your hands. You look up to the basket, and then return your gaze downwards to the ball in your hands; you bounce it once, twice, hearing the thud as the ball contacts the floor. You then pause, looking up again to the basket. You feel your legs and arms flex as you begin the shot and then extend as the shot proceeds, your wrist finally flexes downward as you add backspin to the ball as it leaves your hand, on its way to the basket. But what is the outcome of this imagined action? Because we control and thus can manipulate the image, many would argue the ball would ‘swish’ through the net, exactly as one would have intended it to do. Contemporary research into action imagery however suggests that in imagined actions, like in actual actions, errors occur. Thus, in action imagery, the ball may not always swish through the net but may sometimes hit the backboard or ‘clank’ off the rim, falling to the ground without going through the hoop. Here, we detail why this latter view, that errors occur in action imagery, seems likely.

Action imagery (often also referred to as motor imagery), is the mental simulation of an action without actual movements (Decety et al., 1989; Jeannerod, 1994). Therefore, by definition, no actual action consequences occur. However, as we will argue below, imagined action consequences may replace actual action consequences at least to a certain degree, serving a similar function as actual action consequences and facilitating performance improvements akin to action execution. Below we will outline a framework for how action consequences can be simulated and how errors are detected in action imagery. We will further review evidence from the action imagery literature to support this position.

### Action consequences in action execution

Human actions are goal-oriented. When one performs an action, one intends to produce perceivable consequences (Prinz, 1997). Distal action consequences occur in the environment, like a basketball ‘swishing’ through the net after a free throw. Proximal action consequences occur on the own body, like the kinesthetic and proprioceptive sensations of the legs and arms flexing and extending when shooting a basketball and feeling how the ball leaves the fingers.

Actual actions do not always result in the intended consequences, sometimes errors occur. Such discrepancies between the intended and the actual action consequences are critical for learning of motor skills (Wolpert et al., 1998; Wulf & Shea, 2004), as they provide feedback about the adequacy of the action that was produced (Thoroughman & Shadmehr, 2000). This feedback enables error detection and subsequent error correction. Further, during the progress of an action, action consequences provide information about the way the action must continue to achieve the intended goal, for instance, in the face of unanticipated perturbations (Shadmehr & Mussa-Ivaldi, 1994).

Here, we describe a *computational model of action control* (e.g., Blakemore et al., 2002; Wolpert & Flanagan, 2001; Wolpert & Kawato, 1998; Wolpert et al., 2001) that captures the role of action consequences for action control. Afterward, we adapt the model to describe processes during action imagery (Davidson & Wolpert, 2005).

According to this computational viewpoint intended action effects (e.g., wanting to shoot the basketball into the basket), anticipated action effects (e.g., the expectation that the ball will set the net into motion when the ball passes through the net), perceived action effects (e.g., watching someone else who shoots the ball into a basket as in action observation, see Eaves et al., 2022), and affordances (e.g., seeing the basket) have the potential to activate the motor commands which usually lead to those effects or match the affordance of perceived objects. The computation of the motor commands is performed by inverse models, i.e., models that compute the motor commands based on those effects or affordances. Once motor commands are activated, two things happen. The motor command is sent onward until specific effectors are activated, and a movement is realized. Note that modulation or inhibition of a motor command is still possible once it is created, otherwise one would constantly react to affordances in the environment. Further, an efference copy of the motor command is made and sent to other brain regions. The efference copy is used by forward models to predict the consequences of the action on the body and on the environment (predicted effects). Predictions derived from forward models play an important role in motor control. Because actual feedback is often too late to guide actions, our brain often uses predicted effects to evaluate the appropriateness of actions (Wolpert et al., 2001). This evaluation is based on a comparison of predicted and intended action effects. If necessary, discrepancies between intended and predicted effects (errors) may even be corrected before an action is (fully) executed. When the action is executed, actual action effects become available, which are compared to predicted as well as intended/anticipated effects. Motor learning is conceptualized as the acquisition of inverse and forward models and their optimization, which results in successively lower discrepancies between intended, predicted and actual effects after repeated action execution (Wolpert et al., 2001). In our view, intended, predicted, and actual effects are represented in a perceptual format, similar to what is assumed in ideomotor theories (e.g., Hommel et al, 2001; Prinz, 1997).

### Action consequences in action imagery: simulation and inhibition

**Fig. 1:**
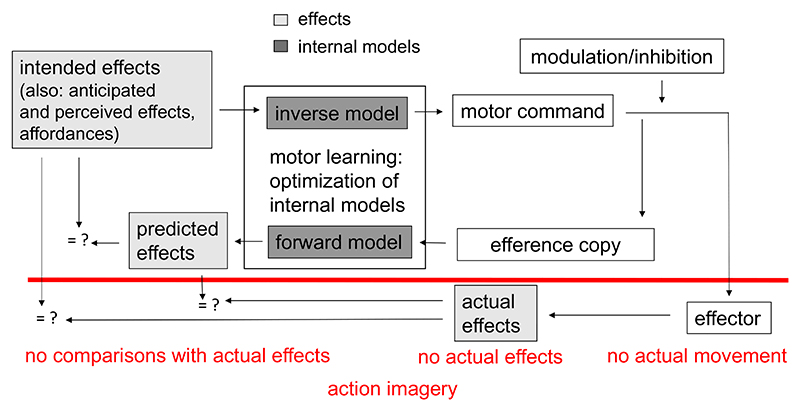
Model of action control (adapted and modified from Blakemore et al., 2002; Dahm & Rieger, 2019a, 2019b). Intended action effects (e.g., wanting to shoot the basketball into the basket), anticipated action effects (e.g., the expectation that the ball will set the net into motion when the ball passes through the net), perceived action effects (e.g., watching someone who shoots the ball into a basket), and affordances (e.g., seeing the basket) have the potential to activate the motor commands which usually lead to those effects or match the affordance of perceived objects using inverse models. After its activation, the motor command is sent to the effectors. Before it reaches the effector, some modulation or inhibition of the motor commands occurs, otherwise one would constantly react to affordances. Further, when a motor command is sent to the effectors, an efference copy is made. The efference copy is used by forward models to predict the consequences of the action on the body and on the environment (predicted effects). Three types of comparisons (indicated by = ?) can take place in executed actions to evaluate the appropriateness of an action and to detect errors: comparisons of intended and predicted effects, comparisons of actual and intended effects, and comparisons of actual and predicted effects. In action imagery, because no actual movement occurs, only the comparison between intended and predicted effects takes place. Motor learning is conceptualized as the acquisition of inverse and forwards models and their optimization, which results in successively lower discrepancies between intended, predicted and actual effects after repeated action execution

Above, we defined action imagery as an internal simulation of actions without actual movements. Such a description implies an embodied cognition view on action imagery (Iachini, 2011), in which action imagery includes a simulation of bodily states (Davidson & Wolpert, 2005; Grush, 2004). The computational model outlined above can be adapted to model how such a simulation takes place in action imagery (see Fig. 1).

Essentially, during action imagery, familiar actions are imagined based on memories of the actions (Annett, 1996) or, from a computational viewpoint, based on internal models for the actions (Davidson & Wolpert, 2005). Thus, if the action is familiar, established internal models can be used for action imagery. If the action is unfamiliar, action imagery may rely on internal models for other, similar actions (Rieger, 2012). Importantly for the present context, according to the computational model, action consequences can be predicted by forward models during action imagery (cf. Dahm & Rieger, 2019a, 2019b; Kilteni et al., 2018; Rieger et al., 2011). But can we take this for granted?

If we assume that motor commands are generated during action imagery, the question arises how the execution of actions is prevented. Sometimes, action imagery is conceptualized as a weaker form of action execution (Guillot et al., 2012; Jeannerod, 2001). This means that motor commands remain subthreshold and the necessity for active inhibition does not arise. However, there is evidence that active inhibition indeed takes place during action imagery (Bart et al., 2021a, 2021b, 2021c; Guillot et al, 2012; Rieger et al., 2017). If consequences of the imagined action on the body and on the environment are simulated during action imagery (cf. Dahm & Rieger, 2019a, 2019b; Kilteni et al., 2018; Rieger et al., 2011), inhibition may block motor commands after the generation of an efference copy. However, others propose that inhibition in action imagery sets in earlier (Solomon et al., 2019), prior to the generation of an efference copy, which may prevent its generation (Berthoz, 1996).

Different inhibitory mechanisms, operating at different levels of the central nervous system, contribute to prevent actual actions in action imagery (Bart et al., 2021a, 2021b, 2021c; Guillot et al., 2012; Rieger et al., 2017). Global inhibition (inhibition of all motor commands) and selective effector-specific inhibition (inhibition of the effector used during action imagery) contribute to action imagery. Global inhibition consists of two different processes (Bart et al, 2021a, 2021b, 2021c). Tonic global inhibition acts proactively and is an internally generated overall readiness to prevent actual actions. It is implemented in contexts in which one expects that a movement shall be imagined. Phasic global inhibition is externally triggered and implemented as a response to a certain event, for instance, a stimulus indicating that an action shall be imagined. Finally, effector-specific inhibition, i.e., selective inhibition of solely the effector(s) used during action imagery is similarly implemented as a response to a certain event, e.g., a stimulus. Those inhibitory mechanisms are not necessarily mutually exclusive. They operate together, complementing each other, but they may differ in their relative importance in different contexts (Bart et al., 2021a, 2021b, 2021c; Guillot et al., 2012). For instance, phasic global inhibition and effector-specific inhibition depend on the amount of tonic global inhibition that already contributes to action imagery (Bart et al., 2021b, see also (Bart, 2021a, 2021c; Rieger et al., 2017).

What does inhibition mean for the internal prediction of action consequences? The crucial question is, at what time point inhibitory mechanisms set in during action imagery. Does inhibition occur prior to or after the generation of an efference copy? Bart et al. (2021c) speculate that the point in time at which inhibition sets in differs between different forms of inhibition. Phasic global inhibition may act fast and temporarily inhibit all actual actions at a very early stage during action imagery (cf. Solomon et al., 2019). It may act at a point in time at which specific effectors have not yet been selected and thus prior to the generation of the efference copy. Effector-specific inhibition sets in later, after the motor command is generated and an efference copy is created (cf. Coxon et al., 2007). Effector-specific inhibition then blocks motor commands before they reach the motor apparatus.

Thus, it seems likely that an efference copy of the motor commands is generated in many instances of action imagery, even though no actual actions occur. This efference copy can be used by forward models to predict the end state of the body after the imagined action as well as proximal and distal consequences of the imagined action (Dahm & Rieger, 2019a, 2019b; Davidson & Wolpert, 2005; Grush, 2004; Kilteni et al., 2018; Miall & Wolpert, 1996). For instance, in dart throwing, one may imagine the exact landing position of the dart on the dartboard (Dahm & Rieger, 2019b). Thus, during imagined actions, a simulation of the action occurs by recruiting processes which are similar to the processes during executed actions. The simulation of action consequences is thereby based on the predictions derived from forward models.

### Action consequences in action imagery: empirical results

When one performs an action, feedback about the action consequences is essential (Thoroughman & Shadmehr, 2000; Wolpert et al., 1998; Wulf & Shea, 2004): *Monitoring action consequences* informs about (a) the progress of an action (e.g., the flexion and extension of the legs and arms during a basketball shot), (b) whether it is necessary to modify the action to achieve the intended goal (e.g., a change of the action when an opponent is getting into a blocking position for the throw), (c) when the next action element should be initiated (e.g., the downward flexion of the wrist after the legs have been fully extended), and (d) whether the action is complete and successful (e.g., whether the ball swished through the basket’s net).

In action imagery, the *lack of actual action consequences* has often been regarded as an essential factor that contributes to differences between action imagery and action execution (Campos et al., 2009; Rieger & Massen, 2014; Rieger, et al., 2011). However, this does not mean that there is no information at all about the progress of the action in action imagery. As outlined above, forward models may predict action consequences. The question arises, whether predicted action consequences can (at least partly) compensate for the lack of actual action consequences to monitor actions in action imagery.

Action consequences can be distal effects one produces in the environment, e.g., a thud occurs when the basketball bounces at the ring, or proximal effects on the own body, e.g., feeling the flexion and extension of the legs and arms during a basketball shot. They can also consist of a change of one’s own position in the environment, e.g., when a basketball player moves around the court, their own position relative to the environment changes constantly while the environment itself remains unchanged.

It appears to be difficult to adequately represent *changes of one’s own position in the environment* during action imagery (Campos et al., 2009; Klatzky et al., 1998). For instance, Campos et al. (2009) asked participants to continuously point at a certain location during imagined and actual walking. They showed that the pattern of pointing was different in action imagery and action execution, indicating that participants did not adequately update their position during imagined walking. However, it is not clear whether inadequate internal prediction processes and/or a conflict between the actual body position (which remains unchanged in action imagery) and the imagined body position contribute to this finding.

How well can participants imagine ongoing effects in the environment? Rieger and Massen (2014) asked participants to execute and imagine coloring rectangles with a thick and a thin pen. In two experiments, participants were able to represent the effects of the characteristics of the pen in action imagery, that is, coloring was faster with a thick than with a thin pen. However, action imagery durations were shorter than action execution durations, indicating that participants may not have correctly predicted the effects of their coloring movements. Participants either imagined having colored more of the area than they actually did or did not adequately monitor which areas were already colored and which were not. Nevertheless, a similar effect was obtained when participants performed action execution without visual feedback. Thus, when no actual distal action feedback is available, predictions of this feedback may be imprecise regardless of whether the action is executed or imagined.

An important function of action consequences is the *detection of action errors*. Action errors provide feedback about the adequateness of an (ongoing) action and are crucial for learning (Thoroughman & Shadmehr, 2000; Wolpert et al., 1998; Wulf & Shea, 2004). In executed actions, action errors can be detected by three mechanisms: (1) a comparison between actual action effects and intended action effects, (2) a comparison between actual action effects and predicted action effects, and (3) a comparison between intended action effects and predicted action effects. Only the third error detection mechanism can operate in action imagery. In executed actions, this mechanism enables one to detect errors even before the action is fully executed (especially in skilled actions), even though they may still be committed (Dahm & Rieger, 2023, Maidhof, Rieger, Prinz & Koelsch, 2009, Rabbitt, 1978). If forward models act as predictors for action consequences in action imagery (e.g., Courtine et al., 2004; Grush, 2004; Rieger et al., 2011), at least some action errors should become evident when the motor command, which is specified by the inverse model and fed into the forward model via the efference copy, is not optimal for the intended effect. For instance, a basketball player may imagine that the ball hits the rim instead of passing through the net. Error detection in action imagery then occurs by comparing the intended and predicted effects.

Actions that are particularly well suited to study action errors are speaking and typing, because errors are frequently committed in those actions. Not all *types of errors* which occur during overt speech (errors due to lexical bias and phonemic similarity) occur in inner speech (only errors due to lexical bias occur, Oppenheim & Dell, 2008, 2010). Inner speech can be regarded as the imagined action of speaking. This indicates that during imagined speaking errors on a lexical-phonological level are adequately represented, but errors on an articulatory-feature-processing level are not (Oppenheim & Dell,). Similarly, in typing, errors can result from failures in different processes (e.g., Grudin, 1983; Logan, 1999). Most processes in typing either relate to higher-order planning or to motor command generation. It has been observed that the detection of higher-order-planning errors does not significantly differ between action imagery and action execution of typing. However, less motor command errors are reported in action imagery than in action execution (Dahm & Rieger, 2019a; Rieger et al., 2011). Thus, only errors that occur in advance to internal modeling are equally observed in action imagery and action execution. However, the reduced detection of motor command errors in action imagery was partly, though not completely, explained by the lack of distal action consequences on the screen, showing that this effect may not be specific to action imagery. Further, it is important to note that some motor command errors are reported in action imagery, indicating that at least in some instances those errors are detected by a comparison of intended and predicted action consequences in action imagery.

Further tasks which are well-suited to investigate errors, or rather *deviations from optimal performance*, are target aiming tasks without time pressure, such as playing darts. In darts, accuracy based on the darts’ final positions on the dartboard is continuous and two-dimensional. Several accuracy measures can therefore be calculated: the mean distance to the target, the consistency across throws, and the bias to systematically deviate from the target in a certain direction (Hancock et al., 1995). In dart throwing, participants should be aware of the approximate distance, as it is visible at each single throw. However, for consistency and bias, information over several throws must be accumulated and participants are therefore less likely to be aware of their own consistency and bias. Thus, the latter two accuracy measures might be particularly suited to inform about internal prediction of action consequences. Participants report different final positions in action imagery of dart throwing, which is a first indication that participants internally predict the consequences of their imagined throws (Dahm & Rieger, 2019b). However, similar to the results in typing (Dahm & Rieger, 2019a; Rieger et al., 2011), participants performed dart throws more accurately in action imagery than in action execution, indicating that the actual extent of deviations from optimal performance is not entirely represented in action imagery. Nevertheless, significant correlations between action imagery and action execution in consistency and bias were obtained (Dahm & Rieger, 2019a). This indicates that participants indeed performed a simulation, because these variables are supposedly outside of participants’ conscious awareness.

Beyond empirical work showing that errors are committed during action imagery, studies show motor learning after *action imagery practice* (for a recent review see Toth et al., 2020). This suggests the presence of a mechanism for the detection and subsequent correction of errors akin to that occurring in learning via action execution. Indeed, while inferior to learning resulting from action execution, learning via action imagery has been demonstrated for a variety of tasks ranging from key presses in sequence learning to whole body movements, consisting of familiar action elements like key presses but also novel movements (e.g., Allami et al., 2008; Dahm et al., 2022; Driskell et al., 1994; Kraeutner et al., 2016; Simonsmeier et al., 2020, for a review see Toth et al., 2020). In many tasks, perceptual–cognitive learning (for instance, learning a sequence of response locations), rather than motor learning (for instance, learning a sequence of movements), seems to dominate action imagery practice (Dahm et al., 2022; see Frank et al., 2023, for a detailed discussion of this issue).

However, if error detection is essential for motor learning in action execution practice, it seems difficult to conceive how action imagery practice could result in learning without similar processes in both types of learning. Indeed, it has been shown that effector-dependent representations (of a sequence), reflecting motor learning, do develop in action imagery practice. However, they develop later than in action execution practice (Dahm & Rieger, 2023). One explanation for this finding is that effector-dependent representations may be optimized due to comparisons of predicted action effects and intended action effects. Because predicted action effects can additionally be compared with actual action effects in action execution practice, but not in action imagery practice, effector-dependent representations take longer to develop in action imagery practice.

In their work, Ingram et al. (2018) asked participants to observe and reproduce a kinematically complex movement pattern at varying speed in action execution practice or action imagery practice. A third group (perceptual control) only observed the movement patterns, reporting the number of direction changes to ensure they were attending to the task. As such, this latter group served to account for the perceptual, as opposed to motoric, elements of learning the movement pattern. Varying speed allowed for the assessment of the speed–accuracy function to investigate the quality of movement execution. Over five sessions, action imagery practice was found to be inferior to action execution practice. However, performance in the action imagery practice group was superior to the performance of the perceptual control group. These results suggest that even though actual action effects (i.e., sensory feedback) are not available in action imagery practice, the simulation of the action results in a means to reduce the discrepancy between internally predicted and intended action consequences.

Further evidence that participants perform a simulation that includes errors in action imagery arises from recent work using this same task (Ingram et al., 2022). In this study, participants once again observed and reproduced a kinematically complex movement pattern at varying speeds via action execution or action imagery. For both action execution and action imagery, participants rated the accuracy of their performance after each trial. For the action execution group, self-rated accuracy was positively correlated with actual accuracy, with both decreasing as the speed and/or complexity of the movement pattern increased. While participants performing the task via action imagery consistently rated their accuracy higher than the action execution group, self-rated accuracy decreased as the speed and/or complexity of the movement pattern increased, paralleling the findings of the action execution group. These findings indicate that accuracy and in turn the commission of errors in action imagery, are impacted by known drivers of error in action execution.

Taken together, what does the occurrence of action errors in action imagery mean for the prediction of action consequences using forward models? Most importantly, it seems that forward models indeed predict action consequences in action imagery. However, this prediction cannot fully compensate for the lack of actual effects, as action imagery results in fewer action errors than action execution (Dahm & Rieger, 2019a, 2019b; Ingram et al., 2022; Rieger et al., 2011) and performance improvements are lower after action imagery practice than after action execution practice (Driskell et al., 1994; Simonsmeier et al., 2020; Toth et al., 2020). It seems that either forward models do not predict all aspects of an action in action imagery, that forward models are imprecise in action imagery, or that in action imagery error signals (discrepancies between intended and internally predicted action consequences) are not sufficiently monitored (Dahm & Rieger, 2019b; Rieger et al., 2011). In particular, action imagery may require more attention for processes that are automatized in action execution but not in action imagery. This makes action imagery cognitively more demanding than action execution (Glover & Baran, 2017), leaving fewer resources to monitor motor command errors in action imagery. One further explanation is that error signals are computed in action imagery but are partly ignored because participants want to perform well. For instance, in action imagery of playing darts, irrespective of the imagined trajectory of the arm and fingers, one may adjust the imagined dart’s trajectory so that the dart flies toward the bullseye (Dahm & Rieger, 2019a, 2019b). Overall, although predictive mechanisms may be similar in action imagery and action execution, the full extent of deviation from optimal performance and errors is not predicted in action imagery.

### Simulation vs. tacit knowledge

In the previous sections, we argued that during action imagery, movements are simulated leading to predictions about the action consequences using forward models. However, not everyone would agree with this view (e.g., Pylyshyn, 2002). The question of how imagery in general is performed has been discussed for a long time in the so-called imagery debate (e.g., Kosslyn, 2005). The central question of this debate is on what kind of representations the subjective experience of imagining something is based. According to the *propositional view*, imagery is based on abstract, amodal, and arbitrary symbols, i.e., the representations are separate and distinct from the modality in which imagery is performed (e.g., Pylyshyn, 2002). According to the *analog view*, modal systems are used to perform imagery (Kosslyn, 2005). Characteristics of the modality, in which imagery is performed, determine how the content of imagery is represented and processed. The imagery debate, which has for years been discussed against the background of visual imagery, can also be applied to action imagery (see Iachini, 2011, for a discussion).

Proponents of the propositional view would argue that action imagery does not make use of the motor system, but instead is based on abstract knowledge, and that the subjective experience of the imagined action is only an epiphenomenon of other, abstract mental processes (Pylyshyn, 2002). According to this view, action imagery draws on abstract knowledge about the movement and its previous consequences which are stored in memory (Annett, 1996). The subjective experience of mentally performing the action therefore has *no causal relation* to the progress of the mentally unfolding action. For instance, if a basketball player knows that they sometimes do not manage to shoot the ball through the basket, they may intentionally incorporate failures into action imagery, rather than detecting action errors based on a simulation.

In contrast to the propositional view, proponents of the analog view would argue, that, as visual imagery makes use of the visual system (e.g., Kosslyn, 2005), action imagery does make use of the motor system (e.g., Grush, 2004, Iachini 2011). Imagery is not abstract but depends on the modal system used to elicit the imagination. Therefore, characteristics and constraints of the motor system determine how the content of action imagery is represented and processed. To induce action imagery, the motor system is used to perform a simulation of the action, which may imply the prediction of action consequences using forward models. Thus, whether a ball goes through the basket or not in action imagery becomes apparent as the simulation of the action progresses and when forward models can predict more and more precisely what the action consequences will be. Hence, during imagery, one does not know the consequences of the action in advance to the imagery process.

Some authors take a stance in between those views. For instance, in the motor–cognitive model of action imagery, it is assumed that action imagery and action execution are similar during the planning phase of an action but deviate from each other during the execution phase (Glover et al., 2020; Glover & Baran, 2017). In particular, to monitor an unfolding action, actual actions sometimes only require unconscious online control, whereas imagined actions always require conscious cognitive (executive) control. According to this view, action imagery is unable to utilize processes related to forward modeling and consequently the prediction of action consequences.

In our view, many results in the action imagery literature are consistent with the analog view. For instance, the observation that bimanual coordination constraints which emerge during action execution and of which one is hardly consciously aware, are also observable in action imagery (Dahm & Rieger, 2016a, 2016b). In particular, the presented results on action errors in action imagery are incompatible with a strict propositional view and with some assumptions of the motor–cognitive model. For instance, the observation that action errors which are not consciously available are represented in action imagery (Dahm & Rieger, 2019b) speaks against the view that action imagery is an epiphenomenon of abstract knowledge about the action and its consequences and the view that the prediction of action consequences does not take place during action imagery. Further support for an analog view of action imagery is provided by results suggesting that during action imagery practice a simulation of action effects serves to reduce discrepancies between internally predicted and intended action consequences (Ingram et al., 2018). This does not rule out that propositional representations exist in addition to analogous representations (Kosslyn, 1994). Indeed, the analog view does not necessarily rule out the existence of additional propositional representations (a basketball player has knowledge about their skill level), but they argue that propositional representations are not sufficient to explain imagery entirely (e.g., Kosslyn, 1981, 1994). Further, this does not rule out that action imagery requires more cognitive control than action execution (cf. Glover & Baran, 2017). Indeed, it may be more demanding on a basketball player’s working memory to imagine a shot than to execute it. However, the important point is that, based on the evidence presented here, it seems hard to explain action imagery without assuming that internal models perform a simulation of the action and an internal prediction of the action consequences during the imagination of an action.

### Conclusions and perspectives

In the present paper, we conceptualized action imagery as a simulation based on internal models. During that simulation, forward models predict action consequences. The comparison of predicted and intended action consequences sometimes indicates the occurrence of action errors (or deviations from optimal performance) in action imagery. The outlined framework seems suitable to cover a wide range of action imagery phenomena and can explain action imagery practice effects.

Nevertheless, there are still a lot of questions that need to be answered. Does a simulation take place in every instance of action imagery? Is the occurrence of a simulation task-dependent? Bach et al. (2022) argue that action imagery is effect-based, that is, one represents the effects one wants to achieve, but does not perform a simulation using the motor system. Though we would not agree that such a model could account for all instances of action imagery, there might be situations when action imagery is performed solely effect-based, for instance, when an action is unfamiliar. What role do individual differences in imagery ability play? If, in addition to a simulation, abstract knowledge contributes to action imagery, how do they interact with each other? If action imagery is multimodal (see Krüger et al., 2022), how are the modalities combined during the simulation and how do modality specific systems (e.g., kinesthetic, visual, auditory) interact? How can task-dependent differences and individual differences in the use of modalities be incorporated into the outlined framework? Are forward models in action imagery and action execution (partly) used in a different manner to predict action consequences?

Above, we conceptualized the processes as similar in action imagery and action execution. However, it is possible that forward models predicting action consequences during action imagery serve functions beyond those they have during action execution. In particular, predictions derived from forward models may serve to compensate for the lack of actual feedback in action imagery (see Solomon et al., 2022 for a similar idea). Further, one is at least partly consciously aware of the predicted action consequences during action imagery, which may not be the case during action execution. Thus, processing demands in one or several functions (such as working memory, cognitive control and monitoring, and internal prediction) may be higher during action imagery than during action execution.

The investigation of action errors in action imagery is a promising approach to investigate the role of simulation and internal prediction of action effects during action imagery. Established lines of research should be continued to investigate some of the open questions. Further, we need to develop new paradigms to further investigate whether effects that are usually observed when people commit errors in actual actions are observed when errors occur in action imagery. For instance, on the behavioral level, sequential effects such as post-error slowing (Dutilh et al., 2012) might be of interest. On the neurophysiological level, components, such as the error-related negativity, which occur shortly after an error and are associated with predictive error detection mechanisms (Joch et al., 2017), might be of interest. Essentially, we predict that post-error slowing and error-related negativity are observable in action imagery. Such research would contribute to our growing understanding of simulation and internal prediction in action imagery and to understanding how action imagery practice drives improvements in performance.

## Data Availability

Data sharing is not applicable to this article as no datasets were generated or analyzed.
